# Imaging-Guided Atherectomy and PCI in a Young Male with Premature CAD and Extensive Calcification

**DOI:** 10.51894/001c.145819

**Published:** 2025-09-30

**Authors:** Yasir Mehmood, Anthony Teta, Jay Mohan

**Affiliations:** 1 Michigan State University, East Lansing, MI, US College of Osteopathic Medicine; 2 McLaren Health Care/Macomb/Oakland MSU Cardiovascular Disease Fellowship McLaren Macomb Hospital, Mt. Clemens, MI, US; 3 Department of Cardiovascular Medicine McLaren Macomb Medical Center, Mt. Clemens, MI, US

## INTRODUCTION

Coronary artery disease (CAD) in young adults is uncommon but requires high clinical suspicion, particularly in patients with significant family history and risk factors. Intravascular imaging is crucial for guiding complex interventions in heavily calcified lesions. We present a case of a 35-year-old male with symptomatic CAD requiring a staged approach using optical coherence tomography (OCT)-guided rotational atherectomy and percutaneous coronary intervention (PCI).

## CASE DESCRIPTION

A 35-year-old male presented to the emergency department with substernal chest pressure (6/10), radiating to his back, associated with diaphoresis and dyspnea. Symptoms had been intermittent for two days. His medical history included erythroderma, dyslipidemia (noncompliant with statins), diverticulosis, GERD, and anxiety. Family history was notable for premature myocardial infarctions (MI) in both parents.

On arrival, he was treated with metoprolol, intravenous nitroglycerin, and heparin, and was initiated on aspirin and atorvastatin. Coronary angiography revealed severe calcification with an occlusive lesion in the proximal LAD. Due to poor surgical targets and potential healing concerns, a decision was made to defer coronary artery bypass grafting (CABG) and pursue PCI. Initial attempts with noncompliant balloon dilation were unsuccessful due to severe calcification identified on intravascular ultrasound (IVUS), leading to deferral of PCI for planned imaging-guided atherectomy.

The patient was discharged on aspirin, prasugrel, metoprolol, atorvastatin, lisinopril, and nitroglycerin. He returned five days later with atypical chest pain, which was managed conservatively. He subsequently underwent PCI with rotational atherectomy and drug-eluting stent placement in the LAD. OCT was utilized intra-procedurally to optimize stent expansion and apposition.

## DISCUSSION AND CONCLUSION

This case highlights the importance of intravascular imaging in managing complex coronary lesions, particularly in young patients with severe calcification. OCT provided high-resolution imaging, allowing for precise lesion assessment, adequate plaque modification, and optimal stent deployment. The decision for a staged approach with atherectomy was critical in achieving a successful outcome. This case underscores the role of advanced imaging techniques in guiding contemporary PCI and improving procedural success in complex coronary interventions.

